# Impact of the 2023 ACR/EULAR Antiphospholipid Syndrome Criteria on Retinal Vein Occlusion Patients

**DOI:** 10.3390/jcm14082826

**Published:** 2025-04-19

**Authors:** Rafael Gálvez-Sánchez, Zaida Salmón González, Magdalena Fernández-García, Andrea Cerveró Varona, Belén González-Mesones, Marcos López-Hoyos, Víctor Martínez-Taboada, José Luis Hernández

**Affiliations:** 1 Division of Rheumatology, Hospital Universitario Marqués de Valdecilla, Instituto de Investigación Valdecilla, 39008 Santander, Spain; rafaelgalvez97@gmail.com (R.G.-S.); vmartinezt64@gmail.com (V.M.-T.); 2 Division of Internal Medicine, Hospital Universitario Marqués de Valdecilla, Instituto de Investigación Valdecilla, 39008 Santander, Spain; zaida.salmon@scsalud.es (Z.S.G.); magdalena.fernandezg@scsalud.es (M.F.-G.); 3 Division of Ophtalmology, Hospital Universitario Marqués de Valdecilla, Instituto de Investigación Valdecilla, 39008 Santander, Spain; andrea.cervero@scsalud.es; 4 Division of Hematology, Hospital Universitario Marqués de Valdecilla, Instituto de Investigación Valdecilla, 39008 Santander, Spain; belen.gonzalezmesones@scsalud.es; 5 Division of Inmunology, Hospital Universitario Marqués de Valdecilla, Instituto de Investigación Valdecilla, 39008 Santander, Spain; marcos.lopez@scsalud.es; 6 Departamento de Medicina y Psiquiatría, Universidad de Cantabria, 39005 Santander, Spain; 7 Department of Internal Medicine, Hospital Marqués de Valdecilla-IDIVAL, Avda. Valdecilla s/n, 39008 Santander, Spain

**Keywords:** antiphospholipid antibodies, antiphospholipid syndrome, retinal vein occlusion, vascular risk factors, classification criteria

## Abstract

**Background/Objectives:** Retinal vein occlusion (RVO) represents a common ophthalmological disorder that, if untreated, often leads to severely impaired vision. The classic vascular risk factors, aging and glaucoma, represent the core pathogenic factors for RVO. However, antiphospholipid syndrome (APS) has been involved in a non-negligible number of patients with RVO. The main objective of the present study was to assess the performance of the new 2023 ACR/EULAR classification criteria for APS in a cohort of patients with RVO fulfilling the Sydney classification criteria. **Methods:** A prospective study of consecutive patients with RVO diagnosed with APS in a third-level university hospital. The new 2023 ACR/EULAR classification criteria for APS were applied to all patients. Vascular risk factors, the antiphospholipid antibody (aPL) profile, clinical management, and clinical outcomes were assessed and compared between those fulfilling the Sydney and the 2023 ACR/EULAR criteria. **Results:** Sixty-nine RVO-APS patients were included in the study. After applying the new classification criteria, 18 patients (26.1%) did not fulfill the new criteria for APS. Specifically, 17 (24.6%) were excluded due to the new Domain 8 (*p* < 0.001) as they presented only aPL IgM serology, and 1 patient (1.4%) was excluded due to having high venous thromboembolic risk (VTE) with a clinical domain score < 3. Interestingly enough, the presence of high arterial risk (45.1% vs. 50%; *p* = 0.72) was greater than the presence of high VTE (3.9% vs. 5.6%; *p* = 0.99); in both cases, the 51 RVO-APS patients were classified with the 2023 ACR/EULAR criteria, and the 18 cases were excluded according to the new classification criteria. Except for the expected differences in serological domains (Domain 7, *p* < 0.001, and Domain 8, *p* < 0.001), we did not find other significant differences in terms of prognosis or risk of recurrence between both groups of patients. **Conclusions:** The implementation of the new ACR/EULAR 2023 classification criteria for APS resulted in the exclusion of about one out of four previously diagnosed RVO-APS patients. The higher prevalence of manifestations of high arterial risk compared with high VTE among both newly classified and excluded APS patients highlights the importance of monitoring cardiovascular risk factors for both the prevention and the management of potential retinal and cardiovascular events.

## 1. Introduction

Retinal vein occlusion (RVO) represents a common ophthalmological disorder that, if untreated, often leads to severely impaired vision. The classic vascular risk factors and aging represent the core pathogenic factors for RVO [1,2,3]. Therefore, it could be considered a manifestation of systemic atherosclerosis, although other factors such as glaucoma have been involved in its pathophysiology [4]. Additionally, RVO has been associated with systemic comorbidities; increased cardiovascular mortality [5]; hypercoagulability status [6]; drug-induced retinal toxicity; and, more recently, COVID-19 [7].

Three main subgroups of RVO are frequently recognized based on the location of the specific vein occluded (central, hemicentral, and branch RVO); however, the possible arterial involvement should always be considered. This is a consequence of the mechanical narrowing of the veins at the points where they cross the arteries, causing hemodynamic changes, classically explained by Virchow’s triad (hemodynamic changes, vascular endothelial damage, and a hypercoagulable state). In the retina, arteries and veins share a common adventitial sheath and are joined at the junction, where the thin-walled vein passes behind the thick-walled, rigid artery, creating an ideal environment for these changes [8]. As a result, the increased rigidity of the retinal artery associated with aging may increase the compression and risk of vein occlusion at these crossing sites. This explains how atherosclerosis and several other cardiovascular risk factors associated with a higher arterial risk profile (arterial hypertension, diabetes mellitus, hyperlipidemia, and chronic kidney disease) are common in patients with RVO [9,10]. The turbulent blood flow developed at the compression site can cause long-term damage to the vein’s wall structure. This damage can lead to the growth of endothelial cells and changes in the vein’s wall structure, which significantly increases the risk of occlusion at this location [11,12].

A hypercoagulable state has also been involved, as a pathogenic factor, in some patients with RVO [13,14]. Antiphospholipid syndrome (APS) is an autoimmune disease characterized by thrombosis and/or pregnancy morbidity in the presence of persistent antiphospholipid antibodies (aPL) [15,16]. Sydney classification criteria [17] were recently updated in 2023 [18] and included the differential scoring of arterial and venous clinical domains based on a probability of risk with well-defined criteria. As several studies have suggested, aPL could also play a role in the development of RVO, causing a prothrombotic vascular endothelial microenvironment [19]. RVO was more prevalent in patients with aPL than in population-based controls, and a high-risk aPL profile (lupus anticoagulant or double-triple positive serology) was frequent in patients with RVO and APS [20]. Furthermore, risk scores such as the adjusted global antiphospholipid syndrome score (aGAPSS) have also shown an association with the risk of recurrent RVO [21]. According to a study by Sciascia et al. [22], 44.5% of APS patients experience thrombotic events, with venous thrombosis being more common (26.1%) than arterial thrombosis (20%). RVO is considered a rare condition in patients with APS, and there is an ongoing debate about whether ocular symptoms are directly linked to the presence of aPL and what the best treatment plan should be for these patients [14,15,23].

Taking into account these considerations, our study aimed to ascertain the performance of the new 2023 ACR/EULAR criteria for APS in a cohort of patients with RVO and APS defined by the Sydney classification criteria and to compare the prevalence of venous and arterial risk factors in the study population.

## 2. Patients and Methods

### 2.1. Study Participants

RVO patients were studied at the University Hospital Marqués de Valdecilla, a tertiary-care center that serves as a reference hospital for a population of 350,000 inhabitants in northern Spain. All consecutive patients diagnosed with RVO from December 2008 to December 2023 at the Department of Ophthalmology were included in this prospective study. RVO diagnosed by an experienced ophthalmologist, according to clinical, fundoscopic, and angiographic criteria, was also assessed at the Internal Medicine outpatient clinic. During the study period, 69 patients out of a total of 596 RVO patients met Sydney APS criteria.

The Sapporo (Sydney revision) APS Classification Criteria were used to diagnose APS [17]. Later on, the new 2023 ACR/EULAR classification criteria were applied to these patients [18]. The information collected from individual cases has been completely anonymized, and the study was approved by the Ethics Committee of Cantabria (code number 2019.340). Participants gave written informed consent.

### 2.2. Data Collection

Data were collected using a prespecified standardized questionnaire, in a computerized database. We assessed the following clinical variables: age, sex, weight, height, body mass index (BMI), current tobacco use, alcohol intake (>20 g per day), high blood pressure (equal or >140/90 mmHg or being on antihypertensive agents), dyslipidemia (serum total cholesterol or triglyceride levels >230 mg/dL and 150 mg/dL respectively or being on lipid-lowering drugs), diabetes mellitus (according to the ADA criteria) [24], past or present history of thromboembolic disease outside the retina, history of ischemic heart disease, stroke or peripheral arterial disease, type of RVO (central or branch), family history of venous thromboembolism, and prescribed treatments. The presence of manifestations included in the new 2023 ACR/EULAR domains, such as vegetations or cardiac valve thickening, suspected or established microvascular affectation, adverse obstetric outcomes, and thrombocytopenia, were retrospectively recorded.

The SCORE2 and SCORE2-OP were calculated using age, smoking status, systolic blood pressure, and non-HDL-cholesterol [25]. The SCORE2 risk categories can be reduced to three as proposed by the 2021 European Society of Cardiology Guidelines on CV (low to moderate, high, and very high) recommending that different numerical cutoff levels be used according to age groups (<50, 50–69, and ≥70 years of age). SCORE2 estimates an individual’s 10-year risk of fatal and nonfatal CV disease events in individuals aged 40–69 years. For healthy people aged ≥70 years, the SCORE2-OP (older persons) algorithm estimates 5-year and 10-year fatal and nonfatal CV disease events. To compare both populations with equal temporality, only the 10-year risk is used in the SCORE2OP.

A carotid ultrasound examination was used to assess the carotid intima-media wall thickness (cIMT) at least 5 mm below the end of the common carotid artery and to detect focal plaques in the extracranial carotid based on the Mannheim consensus plaque criteria: a protrusion at least 50% greater than the surrounding cIMT, a focal protrusion in the lumen measuring at least cIMT > 1.5 mm, or arterial lumen encroaching >0.5 mm [26,27].

### 2.3. Laboratory Parameters

Blood samples were obtained from an antecubital vein in the morning after a requested 12 h overnight fast (within the week following the first outpatient visit). Routine biochemical parameters were measured by standard automated methods in an ADVIA 2400 Chemistry System autoanalyzer (Siemens, Munich, Germany). We estimated the glomerular filtration rate (eGFR) using the Chronic Kidney Disease Epidemiology Collaboration (CKD-EPI) equations [28]. Regarding coagulation studies, blood samples were collected in vacutainer tubes containing Na citrate 3.2% in 1/9 proportion. After centrifugation (2500 rpm), 1 mL aliquots were stored at −30 °C and tested within 38 days. The hypercoagulability study included platelet count, prothrombin time, activated partial thromboplastin time, fibrinogen, lupus anticoagulant (LA), anticardiolipin antibodies (aCL), and anti-β2 glycoprotein I (aB2GPI). LA was determined with the hexagonal phase phospholipid neutralization test by a coagulometric method and Staclot^®^ LA reagent (DiagnosticaStago, Hong Kong, China). Serum aCL and aβ2GPI were determined using ELISA and following the manufacturer’s instructions (Orgentec^®^, Mainz, Germany). The study also included protein C and protein S levels, antithrombin FVQ506 (FV Leiden), and prothrombin 20210A mutation. Normal values were established according to 100 control patients of the same age range and sex and were as follows: antithrombin, 85–140%; protein C, 85–140%; and protein S, 70–120%. In patients or controls whose initial tests were positive for aPLs, we performed a second test after 12 weeks. If medium or high titers of APLs were detected, the test was considered positive. We performed a third test after another 12 weeks, in participants whose initial and second tests showed some discrepancies and then computed the results of this third test. In cases with positive aPLs, an antinuclear antibody (ANA) test was performed by indirect immunofluorescence. A titer >1/160 was considered as a positive result.

### 2.4. Statistical Analysis

Results were expressed as numbers (percentage), mean ± standard deviation (SD), or median and interquartile range (IQR), according to the distribution of the data tested by the Shapiro–Wilk test. A Student’s *t*-test, Mann–Whitney U test, or one-way ANOVA with Bonferroni correction was used to compare the quantitative variables, and a Chi-squared or Fisher test was used to compare the categorical data. A *p*-value <0.05 was considered statistically significant in all the calculations.

## 3. Results

### 3.1. General Characteristics of the Cohort

We compared the demographic characteristics, cardiovascular risk factors, and main comorbidities in patients who fulfilled the Sydney and 2023 ACR/EULAR classification criteria (Table 1).

The age was slightly higher in patients who did not fulfill the new criteria (73.7 ± 11.8 vs. 68.6 ± 12.9 years; *p* = 0.15). Cardiovascular risk factors were prevalent across all groups, with more than 80% of patients having at least one cardiovascular risk factor (*p* = 0.99). Smoking and diabetes were slightly more frequent in patients fulfilling the new criteria, although without statistical significance (*p* = 0.05 and *p* = 0.18, respectively). Main laboratory parameters were also compared (Supplementary Table S1), and again, we did not find significant differences between both groups.

### 3.2. Impact of the 2023 ACR/EULAR Classification Criteria

**Figure 1:**
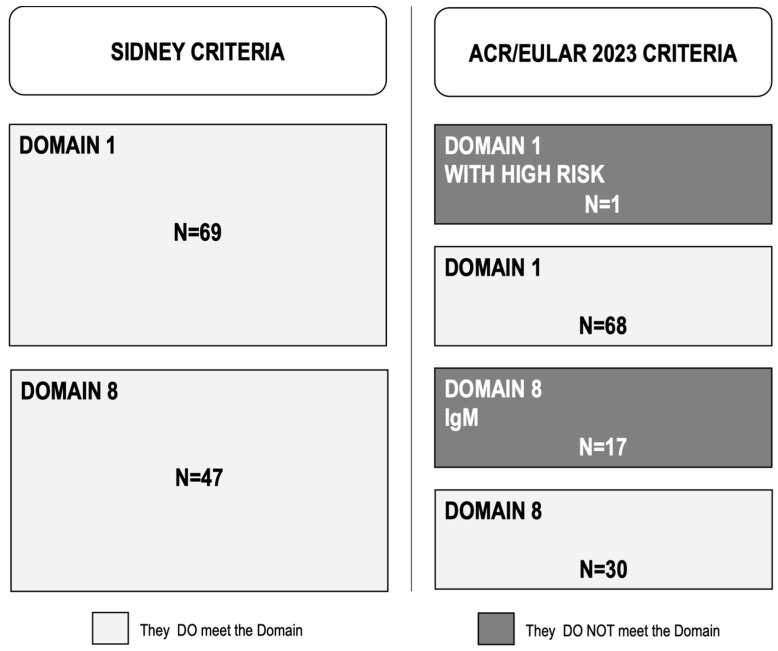
Patients classified as antiphospholipid syndrome by the Sydney and the 2023 ACR/EULAR criteria who meet or did not meet Domain 1 and Domain 8 from ACR/EULAR criteria.

During the study period, 69 RVO consecutive patients classified as APS by Sydney criteria (37 men and 32 women; mean age 69.9 ± 12.8) were assessed (Table 1). After applying the new 2023 ACR/EULAR classification criteria, 18 patients (26.1%) were no longer classified as having APS. As shown in Figure 1, one of these patients did not meet the clinical domains, receiving only one point in Domain 1 (venous thromboembolism) as a high-risk patient and no additional points in the other clinical domains. The other 17 patients were no longer classified as having APS because they received only one point in Domain 8 for having only IgM antibodies for aCL and/or aB2GPI. None of the patients had microvascular or obstetric domain involvement, and only 1 of the 18 declassified patients had cardiac involvement (5.6%) (Table 2).

Regarding the laboratory domains (Table 3), 40 patients (58%) of the 69 classified as APS had a positive LA and therefore met Domain 7 of the ACR/EULAR criteria, with 38 of them (55.1%) having persistent LA positivity. Of the 51 patients classified as APS by the new criteria, 39 had a positive LA (76.5%), thus scoring in Domain 7, with 37 of these patients (72.5%) having persistent LA compared with only 1 patient among the 18 non-classified patients (5.6%), *p* < 0.001. Domain 8 was completed by 47 patients (68.1%) out of 69 from the Sydney group and by 29 patients (56.9%) out of 51 patients who met the ACR/EULAR criteria. All patients (100%) declassified as APS by the new criteria met Domain 8 (*p* < 0.0001). Moderate or high IgM+ (aCL and/or aB2GPI) were found in 8 out of 51 patients (15.7%) who were classified as APS by ACR/EULAR compared with 17 out of the 18 not-classified (94.4%) (*p* < 0.001). High IgG titers (aCL or aB2GPI) were positive in 14 (27.5%) of those who met the new criteria and in none of those who did not (*p* = 0.032). Regarding autoantibody load, double positivity was seen in 6 (11.8%) in the ACR/EULAR criteria group and 11 (61.1%) of those who did not fulfill the new criteria (*p* = 0.0001). Although triple positivity and high-risk aPL profile were seen more frequently in patients who fulfilled the ACR/EULAR criteria, differences did not reach statistical significance (Table 3).

### 3.3. Treatment Before and After Diagnosis

The analysis of treatment patterns in APS patients revealed notable shifts following diagnosis of RVO (Table 4). Before diagnosis, a small number of patients were on low-dose aspirin (LDA), 14.4% under the Sydney Criteria and 11.7% under the 2023 ACR/EULAR Criteria. Those rates dramatically rose after diagnosis to 69.5% and 70.5%. Additionally, statin use also increased from 34.7% before diagnosis to 76.8% after in the Sydney group and from 37.2% to 80.3% in the ACR/EULAR group. The differences did not reach statistical significance.

### 3.4. High-Risk Venous Thromboembolism (VTE) and Cardiovascular Disease (CVD) Profiles

As shown in Table 2, all patients met Domain 1 since RVO belongs to the VTE domain, and three of them had a high risk for VTE (4.3%). Only six of them met Domain 2 (8.7%) for arterial thrombosis (AT). Notably, 32 patients (46.4%) had a high risk for CVD. After applying the new classification criteria, the high-risk VTE profile was 3.9% compared with 45.1% for CVD, although only 11.8% developed manifestations belonging to AT.

### 3.5. aGAPSS

The mean aGAPSS values in the study groups are shown in Supplementary Table S2. The median (interquartile range) aGAPSS values were 8 (7–13), 8 (7–12), and 10.5 (8–13) in the RVO-Sydney group, patients with RVO who met ACR/EULAR criteria, and patients with RVO who did not meet ACR/EULAR criteria, respectively. The aGAPSS was categorized into three risk categories: low (<6 points), medium (between 6 and 11 points), and high risk (≥12 points) [29]. No differences were found between patients who fulfilled or did not fulfill the new criteria in the proportion of patients within each risk category.

### 3.6. SCORE2/OP, Carotid Ultrasound, and Cardiovascular Events

The analysis of SCORE2/OP, carotid ultrasound, and cardiovascular events in patients who fulfilled the Sydney and 2023 ACR/EULAR classification criteria is summarized in Supplementary Table S3. The SCORE2/OP score was slightly lower in patients who met the ACR/EULAR criteria (median (interquartile range): 9.6 (5.9–20.1)) compared with those who did not meet these criteria (14.3 (7.3–22.6)), although this difference was not statistically significant (*p* = 0.36).

Abnormal carotid ultrasound was less frequent in the group that met the 2023 ACR/EULAR criteria (51.1%) compared with patients who did not meet the criteria (68.8%) (*p* = 0.22).

The incidence of previous thromboembolic events, such as VTE, stroke, ischemic cardiopathy, and peripheral ischemic disease, as well as the follow-up thrombotic events, did not show significant differences between patients meeting or not the 2023 ACR/EULAR criteria.

### 3.7. Recurrence of Retinal Vein Thrombosis

The recurrence of RVO in patients who fulfilled the Sydney and 2023ACR/EULAR classification criteria is detailed in Table 5. The total recurrence rate of RVO was slightly higher in patients who met the ACR/EULAR criteria (9.8%) compared with those who did not meet these criteria (5.6%) (*p* = 0.99). The incidence of previous recurrences was higher in patients who met the ACR/EULAR criteria (5.9%) compared with those who did not meet these criteria (0%) (*p* = 0.56). During the follow-up period, the recurrence of RVO was 5.9% in patients who met the ACR/EULAR criteria and 5.6% in those who did not meet these criteria (*p* = 0.99).

## 4. Discussion

The introduction of the 2023 ACR/EULAR classification criteria for APS has significantly impacted the classification of patients, with a relevant number of recent studies published, aimed at validating their specificity and sensitivity in different cohorts of patients, including those with RVO, as shown in the present study.

Foddai SG et al. [30] showed that approximately half of the patients diagnosed with APS according to the Sydney criteria would not meet the new 2023 ACR/EULAR classification criteria, and Lu Q et al. [31], who examined two Asian cohorts, reported a high specificity but lower sensitivity compared with the Sydney criteria, especially at the expense of the obstetric morbidity and the presence of IgM isotype antibodies. Similar findings were observed in the study carried out in Turkey [32]. Our results showed that after applying the new criteria, 26.1% of previously classified APS patients were excluded. The majority of them (17 of 18) were excluded due to Domain 8 of the new classification criteria as they only reached one point with the new scoring system. All these patients carried persistent IgM isotype antibodies at a moderate-high titer, and 11 out of these 17 patients showed double positivity for IgM aCL and aB2GPI antibodies. Interestingly enough, and despite the advanced age of the study population, only one patient did not fulfill the new criteria because of a high VTE risk. Furthermore, and apart from the obvious differences in the serological domains, patients who met the new ACR/EULAR criteria did not differ in other general characteristics, laboratory tests, treatment needs, risk of vascular complications, or recurrences, suggesting that the new criteria do not contribute to characterize a different or more specific subtype of the disease.

This reclassification has clinical implications. Patients excluded from the APS classification under the new criteria may still be at risk for thrombotic events but may not receive the same level of monitoring or treatment as those who meet the new criteria [33]. This highlights the need for a more detailed approach in managing patients with borderline or excluded APS diagnoses to ensure they receive appropriate care based on their individual risk profiles. This is especially true when patients were mostly excluded based on a serologic profile, although a significant proportion of them were double positive for IgM aCL and aB2GPI antibodies. It is important to emphasize the therapeutic consequences this consideration may have. Patients with a highly suggestive clinical and serological picture of APS should receive appropriate treatment and monitoring based on experienced clinical judgment, regardless of the new classification criteria [34].

This study highlights the significant association between RVO and arterial risk factors, suggesting that RVO may often be more accurately characterized as an “arterial” rather than a venous disorder. This is particularly evident in patients with underlying atherosclerosis and other cardiovascular risk factors. The majority of patients (85.5%) included in the present study had at least one cardiovascular risk factor, and nearly half of them (46.6%) had a high risk for arterial thrombosis compared with only 4.3% with high VTE risk. Notably, in the vast majority of RVO-APS patients, low-dose aspirin associated with antihypertensive and lipid-lowering drugs seems to be efficient enough to prevent new thrombotic or vascular disorders after the first RVO episode. Studies such as those by Wong et al. [27] have shown that effective management of hypertension and lipid levels significantly reduces the risk of recurrent RVO [35]. As previously suggested by Hernández et al. [20], it is possible that RVO-APS represents an organ-specific manifestation of APS, where treatments with oral anticoagulants are not as necessary as in patients with thrombotic events in other locations.

Several studies highlight the pathophysiological mechanisms linking arterial disease to RVO [3,36]. A study by Karia et al. [37] demonstrated that patients with RVO had significantly higher incidences of hypertension and hyperlipidemia compared with controls, reinforcing the role of arterial factors in RVO pathogenesis. Dodson et al. [38] analyzed 61 patients (26 with central and 35 with branch-type RVO) with single RVO and 17 with recurrent disease. They found that hyperlipidemia (47% vs. 33%), hypertension (88% vs. 48%, *p* < 0.01), and alcohol intake >7 g/day (47% vs. 13%; *p* < 0.01) could be risk factors for relapsing RVO. Lower serum HDL cholesterol levels (1.24 ± 0.3 vs. 1.46 ± 0.3 mmol/L; *p* < 0.02) and increased systolic blood pressure (175 ± 30.2 vs. 156 ± 26.4 mmHg; *p* < 0.01) were also observed in relapsing patients compared with single RVO. Interestingly enough, in our population, intensive treatment of cardiovascular risk factors, especially hypertension and dyslipidemia, as well as the addition of LDA, was accompanied by a very low recurrence rate.

Moreover, the increased rigidity of the retinal artery associated with aging further contributes to the risk of vein occlusion at arteriovenous crossings. This highlights the importance of monitoring and managing systemic arterial conditions to prevent RVO. Interventions targeting hypertension, hyperlipidemia, and other cardiovascular risk factors may therefore be crucial in reducing the incidence or the recurrence of RVO [5,39].

Furthermore, the role of hypercoagulability in RVO has been a subject of considerable research. While some studies have shown conflicting results, there is evidence to suggest that conditions such as hyperhomocysteinemia and the presence of aPL are associated with an increased risk of RVO. This supports the view that systemic inflammatory and thrombotic conditions, typically associated with arterial disease, play a significant role in the pathogenesis of RVO [1,40]. V. Pengo et al. [33] found that patients with RVO had higher levels of aPL and homocysteine, indicating a prothrombotic state contributing to RVO development [41]. In this regard, Hernández et al. demonstrated a significantly higher prevalence of aPL in RVO patients compared with controls (10% vs. 4.3%; adjusted OR 2.47, *p* = 0.009), reinforcing the hypothesis that acquired thrombophilia, particularly APS, may contribute to the pathogenesis of RVO [20].

Our study has the limitations of observational studies and the small number of included RVO-APS patients, although it is one of the largest studies on this topic. As an observational study, no causality can be inferred. While subgroup analyses can provide valuable insights, those involving small sample sizes are prone to significant reliability issues and should be approached with caution. The prevalence of APS in our RVO cohort is approximately 11.5%, which is consistent with previously reported data in other cohorts. Janssen et al. reported a 5% prevalence of APS in patients with RVO [40], while Hernández et al. identified a 10% prevalence of aPL in RVO patients [20]. Although this is a prospective cohort study, not all patients were evaluated by echocardiogram, and it is possible that the skin manifestations included in the new criteria were also not properly evaluated. The main strengths of the study are that it represents a prospective and real-world assessment of a large cohort of patients with RVO in the outpatient setting and a close, long-term follow-up, which allows assessing the number of RVO relapses or thrombotic or vascular events outside the retinal vessels. Furthermore, our study not only includes a detailed analysis of the cardiovascular risk factors but also evaluates this risk by validated scores and carotid ultrasound, which allows an adequate assessment of the arterial thrombotic risk of the patients.

In summary, the implementation of the new 2023 ACR/EULAR classification criteria for APS resulted in the exclusion of about a quarter of previously diagnosed RVO-APS patients, mostly due to the serological profile. In our opinion, a significant percentage of excluded patients could be considered as a high-risk serological profile (persistently elevated aCL and aB2GPI IgM antibodies). The higher prevalence of manifestations of high arterial risk compared with high VTE among both newly classified and excluded APS patients highlights the importance of monitoring cardiovascular risk factors for both the prevention and the management of potential retinal and cardiovascular events. These findings should encourage clinicians to integrate a more comprehensive evaluation of arterial risk factors into their decision-making process, ensuring that patients receive individualized management strategies that address both thrombotic and cardiovascular risk. Future studies are needed to reinforce these findings and further clarify their clinical implications.

## Figures and Tables

**Table 1:** Demographic characteristics, cardiovascular risk factors, and main comorbidities in patients who fulfilled Sydney and 2023 ACR/EULAR classification criteria.

	Sydney Criteria *n* = 69	2023 ACR/EULAR Criteria		
		Yes *n* = 51	No *n* = 18	*p*
**Age, *yrs ± SD***	69.9 ± 12.8	68.6 ± 12.9	73.7 ± 11.8	0.15
**Sex (male), *n (%)***	37 (53.6)	28 (54.9)	9 (50)	0.72
**Cardiovascular risk factors, *n (%)***	59 (85.5)	43 (84.3)	16 (88.9)	0.99
*** -Obesity***	26 (37.7)	19 (37.3)	7 (38.9)	0.90
*** -Smoking***	10 (14.5)	10 (19.6)	-	0.05
*** -High blood pressure***	46 (66.7)	33 (64.7)	13 (72.2)	0.56
*** -Diabetes***	20 (29)	17 (33.3)	3 (16.7)	0.18
*** -Dyslipidemia***	48 (69.6)	35 (68.6)	13 (72.2)	0.78
**Glaucoma, n *(%)***	16 (23.2)	11 (21.6)	5 (27.8)	0.75

SD, standard deviation; n, number.

**Table 2:** Clinical domains according to the 2023 ACR/EULAR classification criteria.

	Sydney Criteria *n* = 69	2023 ACR/EULAR Criteria		
		Yes *n* = 51	No *n* = 18	*p*
**D1: Macrovascular (VTE), *n (%)***	69 (100)	51 (100)	18 (100)	-
*** -With a high-risk VTE profile***	3 (4.3)	2 (3.9)	1 (5.6)	0.99
**D2: Macrovascular (AT), *n (%)***	6 (8.7)	6 (11.8)	-	0.33
*** -With a high-risk CVD profile***	32 (46.4)	23 (45.1)	9 (50)	0.72
**D3: Microvascular, *n (%)***	-	-	-	-
**D4: Obstetric, *n (%)***	-	-	-	-
**D5: Cardiac valve, *n (%)***	1 (1.4)	-	1 (5.6)	0.26
**D6: Thrombocytopenia, *n (%)***	6 (8.7)	5 (9.8)	1 (5.6)	0.99

D, domain; VTE, venous thromboembolism; n, number; AT, arterial thrombosis.

**Table 3:** Serological domains according to the 2023 ACR/EULAR classification criteria.

	Sydney Criteria *n* = 69	2023 ACR/EULAR Criteria		
		Yes *n* = 51	No *n* = 18	*p*
**Domain 7, *n (%)***	40 (58)	39 (76.5)	1 (5.6)	<0.001
*** -LA+ persistent, n (%)***	38 (55.1)	37 (72.5)	1 (5.6)	<0.0001
**Domain 8, *n (%)***	47 (68.1)	29 (56.9)	18 (100)	<0.001
*** -Moderate or high IgM + (aCL and/oraβ2GPI)***	25 (36.2)	8 (15.7)	17 (94.4)	<0.0001
*** -Moderate IgG + (aCL+ and/or aβ2GPI)***	3 (4.3)	3 (5.9)	-	0.7
*** -High IgG + (aCL+ or aβ2GPI)***	14 (20.3)	14 (27.5)	-	0.032
*** -High IgG + (aCL+ and aβ2GPI)***	5 (7.2)	4 (7.8)	1 (5.6)	0.84
**Combined serology, *n (%)***				
*** -Double+***	17 (24.6)	6 (11.8)	11 (61.1)	0.0001
*** -Triple+***	12 (17.4)	11 (21.6)	1 (5.6)	0.24
*** -High-risk aPL profile***	53 (76.8)	41 (80.4)	12 (66.7)	0.33

n, number; LA, lupus anticoagulant; aCL, anticardiolipin antibodies; aβ2GPI, anti-β2 glycoprotein I antibodies.

**Table 4:** Main treatments in the different study groups before and after diagnosis of retinal vein thrombosis.

	Sydney Criteria *n* = 69	2023 ACR/EULAR Criteria		
		Yes *n* = 51	No *n* = 18	*p*
**Before Treatment**				
**Standard treatment,** *%*				
-LDA	10 (14.4)	6 (11.7)	4 (22.2)	0.488
-Oral anticoagulants	2 (2.9)	1 (1.9)	1 (5.5)	0.999
**Additional treatment,** *%*				
-Statins	24 (34.7)	19 (37.2)	6 (33.3)	0.990
-Hypertension drugs	36 (52.1)	26 (50.9)	10 (55.5)	0.952
**After Treatment**				
**Standard treatment,** *%*				
-LDA	48 (69.5)	36 (70.5)	12 (66.6)	0.990
-Oral antocoagulants	11 (15.9)	8 (15.6)	3 (16.6)	0.999
**Additional treatment,** *%*				
-Statins	53 (76.8)	41 (80.3)	12 (66.6)	0.389
-Hypertension drugs	45 (65.2)	32 (62.7)	13 (72.2)	0.661

LDA, low-dose aspirin.

**Table 5:** Retinal vein thrombosis recurrences in patients who fulfilled the Sydney and 2023 ACR/EULAR classification criteria.

	Sydney Criteria *n* = 69	2023 ACR/EULAR Criteria		
		**Yes** ***n* = 51**	**No** ***n* = 18**	***p***
Total recurrences, *%*	6 (8.7)	5 (9.8)	1 (5.6)	0.99
Previous recurrences, *%*	3 (4.3)	3 (5.9)	0	0.56
Follow-up recurrences, *%*	4 (5.8)	3 (5.9)	1 (5.6)	0.99

One patient had a recurrence previous to the diagnosis and another during the follow-up.

## Data Availability

The original contributions presented in this study are included in the article/Supplementary Material. Further inquiries can be directed to the corresponding author.

## Supplementary Materials

The following supporting information can be downloaded at: https://www.mdpi.com/article/10.3390/jcm14082826/s1, Table S1: Main laboratory parameters in patients that fulfilled Sydney and 2023 ACR/EULAR classification criteria; Table S2: Risk aGAPSS groups according to Sydney and to the 2023 ACR/EULAR classification criteria; Table S3: Cardiovascular events, SCORE, and carotid ultrasound in patients that fulfilled Sidney and 2023 ACR/EULAR classification criteria.
