# Supplementary material for: Impact of the 2023 ACR/EULAR Antiphospholipid Syndrome Criteria on Retinal Vein Occlusion Patients
Source: J Clin Med. 2025 Apr 19;14(8):2826. doi: 10.3390/jcm14082826 (PMC12028246; doi:10.3390/jcm14082826)
Supplement: Supplementary file 1 [file jcm-14-02826-s001.zip › jcm-3522141-supplementary.pdf]

Supplementary Table S1. Main laboratory parameters in patients that fulfilled Sydney and 2023 ACR/EULAR classification criteria.

	**Sydney Criteria N= 69**	**2023 ACR/EULAR Criteria**		
		**Yes *n*=51**	**No *n*=18**	***p***
**Glucose, *mg/dl***	106±32	108±36	99±17	0.14
**HbA1c, %**	6 [5.7-7.3]	6 [5.7-7.2]	6 [5.3-7.2]	0.92
**Total colesterol, *mg/dl***	198±45	199±45	192±46	0.25
**HDL colesterol, *mg/dl***	54±16	52±16	57±15	0.13
**LDL colesterol, *mg/dl***	121±37	123±38	116±36	0.25
**Non-HDL colesterol, *mg/dl***	144±39	148±40	134±39	0.11
**Triglycerides, *mg/dl***	111 [82-148]	115 [91-151]	96 [63-118]	0.055
**Homocystein, *nmol/L***	18.3±6.7	19±7	17±6	0.25

Results are expressed as mean±SD or median [IQR]

Supplementary Table S2. Risk aGAPSS groups according to Sydney and to the 2023 ACR/EULAR classification criteria.

	**Sydney Criteria N= 69**	**2023 ACR/EULAR Criteria**		
		**Yes** ***n*=51**	**No** ***n*=18**	***p***
n, *median [IQR]*	8 [7-13]	8 [7-12]	10.5 [8-13]	0.21
aGAPSSrisk				
-Low Risk	10 (14.5)	8 (15.7)	2 (11.1)	0.93
-Moderate Risk	37 (53.6)	30 (58.8)	7 (38.9)	0.24
-High Risk	22 (31.9)	13 (25.5)	9 (50)	0.1

Supplementary Table S3. Cardiovascular events, SCORE2/OP, and carotid ultrasound in patients that fulfilled Sidney and 2023 ACR/EULAR classification criteria.

	**Sydney Criteria** ***n*= 69**	**2023 ACR/EULAR Criteria**		
		**YES** ***n*=51**	**NO** ***n*=18**	***p***
SCORE2/OP	10.1 [6.6-21.2]	9.6 [5.9-20.1]	14.3 [7.3-22.6]	0.36
Abnormal carotid ultrasound	34 (55.7)	23 (51.1)	11 (68.8)	0.22
**Previous thrombotic events, %**				
-Venous thromboembolic disease	2 (2.9)	1 (2)	1 (5.6)	0.46
- Stroke	5 (7.2)	5 (9.8)	0	0.32
-Ischemic cardiopathy	5 (7.2)	4 (7.8)	1 (5.6)	0.99
-Peripheral ischemic disease	4 (5.8)	3 (5.9)	1 (5.6)	0.99
**Follow-up thrombotic events, %**				
-Venous thromboembolic disease	1 (1.4)	1 (2)	0	0.99
- Stroke	2 (2.9)	2 (3.9)	0	0.99
-Ischemic cardiopathy	2 (2.9)	0	2 (11.1)	0.06
-Peripheral ischemic disease	4 (5.8)	3 (5.9)	1 (5.6)	0.99
